# Irrigation and warming drive the decreases in surface albedo over High Mountain Asia

**DOI:** 10.1038/s41598-022-20564-2

**Published:** 2022-09-28

**Authors:** Fadji Z. Maina, Sujay V. Kumar, Chandana Gangodagamage

**Affiliations:** 1 Hydrological Sciences Laboratory, NASA Goddard Space Flight Center, Greenbelt, MD USA; 2 Goddard Earth Sciences Technology and Research Studies and Investigations, University of Maryland, Baltimore County, Baltimore, MD USA

**Keywords:** Climate sciences, Environmental sciences, Hydrology

## Abstract

Human and climate induced land surface changes resulting from irrigation, snow cover decreases, and greening impact the surface albedo over High Mountain Asia (HMA). Here we use a partial information decomposition approach and remote sensing data to quantify the effects of the changes in leaf area index, soil moisture, and snow cover on the surface albedo in HMA, home to over a billion people, from 2003 to 2020. The study establishes strong evidence of anthropogenic agricultural water use over irrigated lands (e.g., Ganges–Brahmaputra) which causes the highest surface albedo decreases (≤ 1%/year). Greening and decreased snow cover from warming also drive changes in visible and near-infrared surface albedo in different areas of HMA. The significant role of irrigation and greening in influencing albedo suggests the potential of a positive feedback cycle where albedo decreases lead to increased evaporative demand and increased stress on water resources.

## Introduction

Surface albedo, the ratio of the solar radiation reflected from the Earth’s surface to the solar radiation incident upon it, is an essential variable determining the energy balance at the land surface^1,2^, in turn influencing local and global climates. A decrease in surface albedo gives rise to a positive radiative forcing, which can counterbalance the negative radiative forcing created by carbon sequestration^3^ and promote surface warming. Surface albedo also has an influence on the fraction of energy transformed into sensible and latent heat fluxes^4–6^. Variations in surface albedo are driven by changes on the Earth surface (vegetation, snow coverage, soil moisture, etc.), the solar illumination, and the zenith angle^7–12^. Also, vegetation phenology and seasonality of climate^13,14^ exert influences on the albedo changes at longer timescales. Consequently, natural disturbances such as warming and human activities such as deforestation and irrigation could alter the surface albedo^15^, often larger than the biogeophysical mechanisms acting on the radiation budgets at both surface and atmospheric levels^16–20^. Therefore, quantifying the drivers of surface albedo changes can provide critical inferences on land-use change impacts on the radiative forcing.

**Figure 1:**
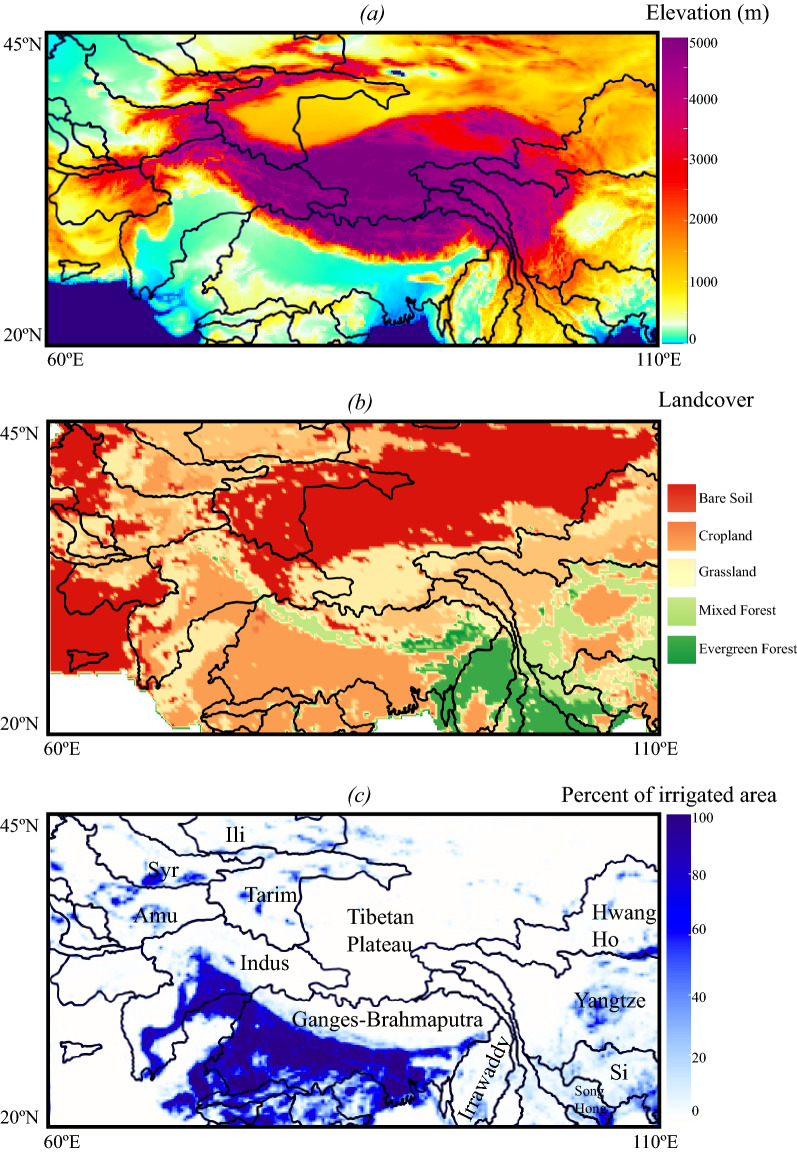
Maps of High Mountain Asia. (**a**) elevation, (**b**) land cover^34^, and (**c**) percent of irrigated areas per pixel^35^. The black lines indicate the limits of the hydrologic basins, and their names are indicated in (**c**).

High-Mountain Asia (HMA, Fig. 1) covering the Tibetan Plateau and its surroundings, consists of densely populated hydrologic basins (e.g., Ganges–Brahmaputra and the Yangtze) serving over a billion people^21–24^. HMA basins play a critical role in sustaining the economy, agriculture, and energy of around 10 countries including China, Nepal, Bangladesh, India, Pakistan, and Afghanistan. Land structure heterogeneity over HMA is tremendous, with elevation ranging from the sea level to the world’s highest point, different climatic conditions (westerlies and monsoons), footprints of human activities, and large variations in land cover types. HMA experiences strong changes in land surface characteristics caused by greening^25,26^, decreases in snow^21^, and irrigation^27^. Because greening changes the optical and structural properties of the vegetation canopy and increases the Leaf Area Index (LAI), it affects surface albedos^28–30^. Decreases in cryospheric storages resulting from warming, changes in precipitation phases, and dust and black carbon deposits also contribute to surface albedo decreases^31–33^. Lastly, significant irrigation activities also influence surface albedo by decreasing ground reflectance and enhancing vegetation growth.

Quantifying the relationships between these cryospheric and biospheric changes and the surface albedo in HMA, where land surface processes play a significant role in the hydrodynamics^36–38^ will provide a better understanding of (1) their impacts on the climate system and water resources^39,40^, (2) the impacts of land surface changes on the radiative forcing, crucial for designing climate change mitigation and adaptation strategies^41–43^, and (3) the contributions of human management to Earth’s warming and/or cooling. Despite the fact that irrigation, warming, and greening are occurring at high rates in HMA, previous studies assessing surface albedo changes were limited to the Tibetan Plateau^44,45^ and the Himalayas^32^.

Satellite remote sensing is an essential technique for estimating surface albedo at various spectral, spatial, and temporal resolutions^46^. Examining the broadband components of surface albedo such as visible radiation (VIS) with a wavelength between 0.3 and 0.7 µm and near-infrared radiation (NIR) with a wavelength between 0.7 and 5.0 µm^46,47^ allows assessing changes in different land surface and vegetation states. For example, vegetation canopies reflect a much larger fraction in the NIR than in the VIS, because plant canopies scatter NIR energy^29,48,49^ whereas the VIS has a stronger negative and positive correlation with the soil moisture and snow cover respectively^19,50–52^. Here, we rely on a partial information decomposition analysis with remote sensing data to quantify the impacts of changes in (1) LAI, (2) soil moisture, and (3) snow cover on the black sky and white sky surface albedos in VIS and NIR broadband over HMA from 2003 to 2020. We use the albedo climatology provided by MODIS V006 (MCD43A3^53^), LAI provided by MCD15A2H Version 6 of MODIS^54^, snow cover fraction provided by MODIS MOD10CM^55^, and soil moisture provided by the European Space Agency Climate Change Initiative (ESA CCI^56^). To disentangle the contributions of the climate (i.e., increases in precipitation) and irrigation to the changes in soil moisture, we use the irrigation datasets provided by^35^ and a precipitation dataset^57^ generated using a localized probability matched method^58^ to blend the best available gridded precipitation products that include the Integrated Multi-satellitE Retrievals for Global Precipitation Measurement IMERG^59^, the Climate Hazards group Infrared Precipitation with Stations CHIRPS^60^, and the ECMWF Reanalysis ERA5^61^.

Based on the analysis of remote sensing datasets of surface albedo and other land surface variables, this study establishes the impacts of irrigation, greening, and warming on the surface albedo, with implications for the development of positive radiative forcing in HMA. Specifically, the study demonstrates that increases in soil moisture in irrigated lands (Ganges–Brahmaputra and Indus) drive the highest decreases in surface albedo. Soil moisture drives the reductions in surface albedo in non-irrigated lands of the Indus and the northern HMA, while the declines in snow cover from warming decrease surface albedo in the Tibetan Plateau. Although warming, dust, and black carbon induced snow cover decreases exert an influence on the surface albedos, these impacts are limited to the water towers and the winter season. Greening enhances NIR and decreases VIS in snow-free forests (e.g., Yangtze). In snow-covered vegetated areas (e.g., the Himalayas, Amu Darya, and Hwang Ho), greening increases both the VIS and the NIR because of the presence of snow. In addition to the established snow albedo feedback, where the reduction in surface albedo over snow-covered areas leads to increased net radiation and sustained melt^62^, the current study outlines another possible positive feedback cycle related to surface albedo. In densely populated areas downstream of the high elevation mountains, irrigation and greening dominate the highest decreases in surface albedo (up to 1%/year), which can lead to increases in evaporative demand and subsequent increased irrigation water use in a positive feedback cycle. The increased stress on the limited water resources in this region from such impacts is a significant concern. These anthropogenic amplifications should be accounted for in climate modeling studies and in designing mitigation strategies for managing the impacts on the water cycle.

## Results

### Surface albedo changes in HMA

**Figure 2:**
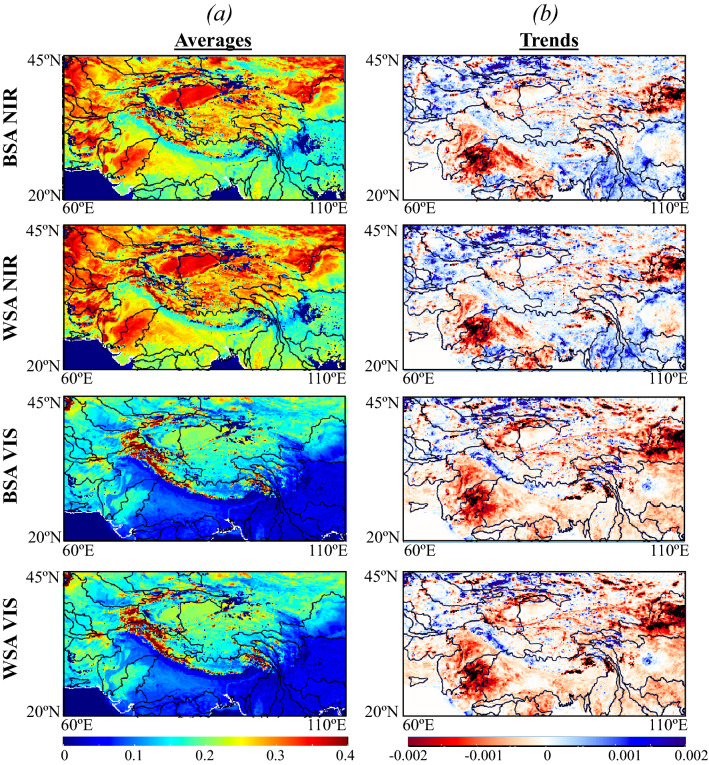
Surface albedo changes and values in High Mountain Asia. Spatial distributions of the yearly (**a**) averages and (**b**) trends from 2003 to 2020 of BSA (Black Sky) and WSA (White Sky) surface albedos in both NIR and VIS wavelengths. Trends were computed using the Mann–Kendall test with a confidence level of 95%.

Forested basins (Yangtze, Si, Song Hong, and Irrawaddy) have the lowest surface albedos due to their dense canopy (Fig. 2a). The highest surface albedos (NIR > 0.35 and VIS > 0.2) are in the Himalayas and the northern HMA due to the presence of snow. In the irrigated lands of the Indus and the Ganges–Brahmaputra, surface albedo values are in between those of forests and bare soil. Surface albedo trends are bidirectional, although the VIS has a decreasing trend almost everywhere (Fig. 2b). The irrigated lands and Hwang Ho have the highest decreases in both NIR and VIS (> − 2.10^−3^/year). The northwestern basins have an increasing trend in NIR (> 10^−3^/year) whereas some areas show no significant trends to increasing trends of VIS. Tarim and the northern HMA are characterized by decreasing trends in NIR (< 10^−3^/year) and VIS (~ − 2.10^−3^/year). Forested basins show an increasing trend in NIR (~ 10^−3^/year) and a decreasing trend in VIS (from 10^−4^ to 10^−3^/year). Previous studies have reported an increasing trend of surface albedo in central Asia^63^ in general and a decreasing trend in the Tibetan Plateau^44,45^ whereas our findings show that these trends are bidirectional in the surface albedos constituents.

Precipitation has a bidirectional trend, some portions of the Indus and the Yangtze basins are characterized by increasing trends, whereas the Ganges–Brahmaputra basin with high precipitation rates has an overall decreasing trend of precipitation although in some areas precipitation sees an increase (Supplementary Fig. 1). The trends of snow cover and LAI are unidirectional with decreasing and increasing trends respectively, whereas the soil moisture has a bidirectional trend (Supplementary Fig. 1). Forests (e.g., the Yangtze basin) have the highest LAI increase and snow cover decrease, yet they depict low surface albedo changes. Moreover, the Hwang Ho characterized by high surface albedos changes is characterized by noteworthy changes in LAI and snow cover. The long-term patterns of surface albedo changes are, therefore, not explained by the changes in vegetation, snow, and soil moisture alone. This following section describes the key land surface processes and their interactions in influencing albedo.

### Drivers of surface albedo changes in HMA

**Figure 3:**
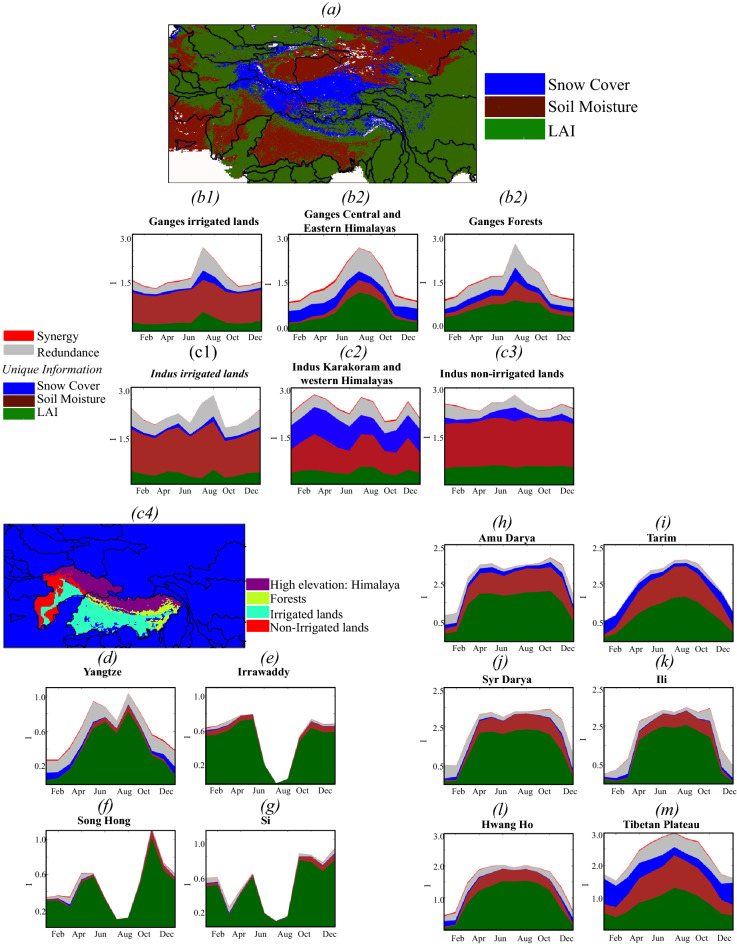
Dominant drivers of the surface albedo changes. (**a**) map of the dominant drivers of the surface albedo changes (i.e., the variable with the highest unique information). (**b**–**m**) Temporal variations of the unique, synergistic, and redundant information of leaf area index, soil moisture, and snow cover about the visible white-sky surface albedo of 16 zones (basin names are indicated in Fig. 1c and subregions c4). Note that y-axis is a stacked graph and is not cumulative.

The partial information framework allows computing the unique information, which is the contribution of a given variable solely (e.g., LAI, soil moisture, or snow cover) to the surface albedo changes. The synergistic information quantifies how the interactions among the different variables contribute to the changes in surface albedo, whereas the redundant information is the non-unique information about the surface albedo encoded redundantly in different variables. Figure 3 which shows the unique, synergistic, and redundant information in various drivers of surface albedo changes, indicates that these factors are spatiotemporally heterogeneous in HMA. Note that we only show the results of the VIS surface albedo because even though the NIR and VIS have different trends, the contributions of the different variables to the dynamics of these surface albedo broadbands remain approximately the same. The unique information from soil moisture, LAI, or snow cover dominates the surface albedo, though in some instances, the redundant information across these variables also becomes important (Fig. 3b–m). The synergistic information across these factors is generally small (Fig. 3b–m). Figure 3a shows the map of the variable with the highest unique information at a given point, i.e., the dominant driver of the surface albedo changes at this point.

Overall, because of HMA’s intense greening^62^, LAI is the main driver of surface albedo changes and dominates the surface albedo changes in forested and northwestern basins. For instance, LAI is the dominant factor with large unique information in the forested areas of Irrawaddy (Fig. 3e), Song Hong (Fig. 3f), and Si (Fig. 3g); this influence progressively reduces depending on the density of the forest canopy (68% in Irrawaddy, 56% in Song Hong, and 32% in Si). The enhanced vegetation growth in these areas originates from an increase in precipitation^62^, and therefore, an increase in soil moisture leads to increasing NIR. Though the unique information of soil moisture is low in these forested areas, it increases during the monsoon.

Due to HMA’s intense irrigation activities, soil moisture is the primary factor influencing surface albedo changes over the irrigated lands of the Indus and Ganges–Brahmaputra. For example, the unique information of soil moisture is three to four times higher than that of LAI and snow cover in the Ganges–Brahmaputra (Fig. 3b) and the Indus (Fig. 3c). As discussed in Section “Surface albedo changes in HMA” and shown in Supplementary Fig. 1, while the soil moisture is characterized by high increasing trends over the irrigated lands of the Ganges–Brahmaputra and the Indus, the precipitation is decreasing in the Ganges–Brahmaputra and shows a low-to-no increase in the low-elevation zones of the Indus basin. As a result, changes in precipitation are not positively correlated to changes in soil moisture (Supplementary Fig. 1c). Therefore, the high increasing trends in soil moisture over these irrigated areas are attributed to irrigation. Soil moisture is also the dominant driver of the changes in surface albedo in the northern area of HMA. As in the irrigated lands, changes in precipitation are not positively correlated to the changes in soil moisture. However, the area experiences a decrease in snow cover (Supplementary Fig. 1) which causes an increase in soil moisture. Compared to different areas of HMA, the redundant information between LAI and soil moisture is non-significant over the Ganges–Brahmaputra (Fig. 3b) and the Indus (Fig. 3c) basins, which reaches a peak in July when the high soil moisture interferes with the soil reflectance by decreasing the VIS component. This is because the soil moisture solely drives the changes in surface albedo, in contrast to the forested basins where it is the greening that stems from increases in soil moisture that has the highest impact on the surface albedo, not the soil moisture.

Because of HMA’s warming^62^, snow is a significant factor in influencing the surface albedo in snow-dominated regions such as the Tibetan Plateau (Fig. 3m), the Karakoram and the western Himalayas (Fig. 3c2), and the central Ganges–Brahmaputra and Eastern Himalayas (Fig. 3b2). There is a seasonality to the snow cover unique information which increases in winter. The contrasting seasonal influences of snow cover, soil moisture, and LAI are also observed in several areas. Because multiple processes, including warming, changes in precipitation, and greening, govern the water and energy balances in the central and eastern Himalayas (Fig. 3b2), Hwang Ho (Fig. 3l), Tarim (Fig. 3i), and the northwestern basins (Amu Darya, Syr Darya, and Ili, Figs. 3h, j and k), surface albedo changes in these zones have multiple drivers whose contributions are seasonally dependent. In the Hwang Ho (Fig. 3l) and Yangtze (Fig. 3d), for example, surface albedo variations are primarily governed by the changes of LAI though snow cover has a non-trivial contribution in winter. Additionally, soil moisture changes in Hwang Ho also affect surface albedo because its vegetated areas (48% of the basin area) are not dense enough to absorb all the solar radiation, so the surface albedo is sensitive to soil moisture. In the central and eastern Himalayas, the unique information of LAI and soil moisture peaks in July (Fig. 3b2). Because the increases in soil moisture are due to snowmelt, the unique information of snow cover and soil moisture have opposite monthly variations. During the growing season, the unique information of LAI is two times higher than that of snow cover and soil moisture combined. The partial information analysis presented here provides important insights about the key processes that drive the surface albedo in HMA basins. Next, we describe the long-term trends and the seasonality of these processes. We regroup the different basins by their dominant driver of the changes in surface albedo.

#### Irrigation induces the highest surface albedo decreases in HMA

##### Drivers of long-term trends

The Ganges–Brahmaputra and the Indus are subject to agricultural activities involving intense irrigation^35^ and groundwater pumping^64^. Irrigated lands occupy 49% of the Ganges–Brahmaputra and 22% of the Indus. They have the highest yearly increases in soil moisture up to 0.03/year and therefore the highest decreases in VIS and NIR in HMA on average equal to − 4.4 10^−4^/year and − 2 10^−4^/year, respectively in the Ganges–Brahmaputra and − 6 10^−4^/year for the VIS and − 2 10^−4^/year for the NIR in the Indus. We note that these high increases in soil moisture originate from irrigation as the precipitation in the Ganges–Brahmaputra and the Indus are decreasing and have a low-to-no increase respectively. As such, the highest yearly decreases in VIS and NIR are from February to June when the soil moisture increases significantly (not shown here). Though not from irrigation, the influence of soil moisture on surface albedo is also seen in other areas (Fig. 3a). In the northern HMA and parts of Indus, soil moisture increases originating from increases in precipitation^57^ (Supplementary Fig. 1) decrease the surface albedo at rates equal to − 2.3 10^−4^/year for the VIS and − 1.6 10^−4^/year for the NIR (Supplementary Fig. 4).

##### Drivers of seasonality

**Figure 4:**
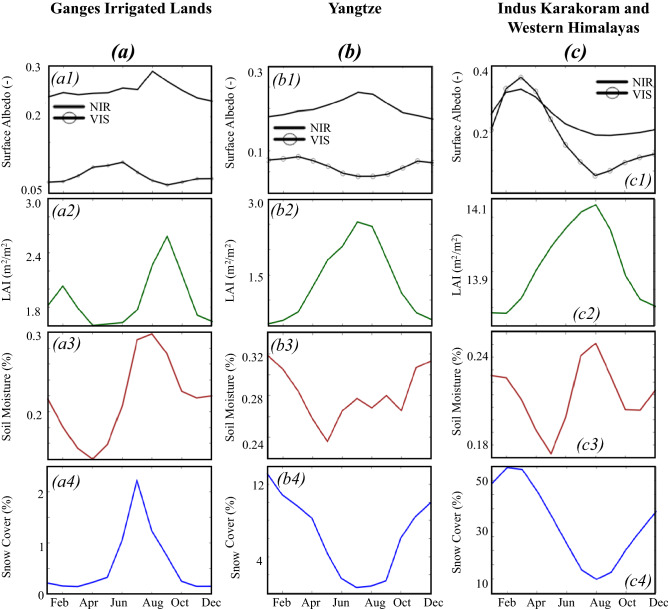
Monthly variations of the averages of surface albedo, LAI, soil moisture and snow cover. (**a**) in a basin where irrigation decreases surface albedo: irrigated lands of the Ganges–Brahmaputra, (**b**) in a basin where greening decreases VIS and increases NIR surface albedo: Yangtze, (**c**) in a basin where changes in snow cover decreases surface albedo: the Karakoram and Western Himalayas in the Indus.

Figure 4a illustrates the seasonality of VIS and NIR surface albedos, LAI, soil moisture, and snow cover in the irrigated lands of the Ganges–Brahmaputra. In the average seasonal cycle, the VIS increases from January to April because of the decreases in LAI and soil moisture. As soil moisture and LAI keep decreasing to reach their lowest values, the VIS reaches its maximum value (0.1) in June. The beginning of the rainy season triggers increases in soil moisture and LAI. Hence the VIS starts to decrease. With the increase in LAI, more incoming solar energy is reflected and scattered by the vegetation canopy, and only a small proportion of the incoming solar radiation reaches the ground^65^. The VIS remains at its lowest value (~ 0.05) for two consecutive months, August and September, when the average LAI and soil moisture have their highest values of 2.5 and 0.29, respectively.

#### Greening decreases the VIS and increases the NIR surface albedo over forested regions of HMA

##### Drivers of long-term trends

Though HMA experiences greening at high rates^27^, LAI only controls surface albedo changes in forests of Ganges–Brahmaputra, Yangtze, Irrawaddy, Song Hong, and Si (Fig. 3a). The Yangtze has one of the highest trends of LAI in HMA (up to 0.02 m^2^m^−2^/year). Nevertheless, the surface albedo trends are low due to their small magnitudes. The VIS and NIR surface albedos have contrasting trends due to the presence of forests. In the Yangtze, the yearly increasing trends of LAI cause the NIR to increase (6 10^−4^/year) and the VIS to decrease (− 2.8 10^−4^/year), consistent with prior studies^66^. Likewise, in the Irrawaddy, greening increases the NIR (up to 4 10^−4^/year) and decreases the VIS (Supplementary Fig. 3a).

##### Drivers of seasonality

Figure 4b depicts the seasonality of VIS and NIR surface albedos, LAI, soil moisture, and snow cover over a forested basin, the Yangtze. Because of its dense canopy and high precipitation (970–1200 mm/year) leading to high soil moisture, annual averages of NIR and VIS surface albedos in the Yangtze are low, equal to 0.275 and 0.187 respectively. In these areas, the patterns of the monthly variations of LAI and soil moisture are similar. The VIS is high in winter due to vegetation senescence with a peak in March, while the NIR component becomes high in summer. The lowest VIS (0.05) is from May to August, when LAI and soil moisture are high, and snow cover low. VIS decreases as the canopy becomes dense and the wetness of the soil increases to dampen the effects of ground reflectance. As the canopy develops, its NIR reflectance increases due to increased multiple scattering^67^. Similar patterns are found in the forested areas of the Ganges–Brahmaputra and Irrawaddy. Ganges–Brahmaputra forests are characterized by a low VIS (0.027, Supplementary Fig. 2b).

#### Snow cover dominates surface albedo changes in high-elevation zones

##### Drivers of long-term trends

Snow cover drives surface albedos changes in the Tibetan Plateau, the Karakoram, and the western Himalayas (Fig. 3a). These areas have an overall increasing trend of surface albedo stemming from an increasing snow cover. However, this increasing trend is only limited to the winter, as surface albedo has a decreasing trend in summer and fall (Supplementary Fig. 7) likely because of dust and black carbon deposits that darken the snow^31,32,68^. Similar patterns are also observed in the Tibetan plateau, where surface albedo decreases because of the decrease in snow cover. The latter has also been attributed to black carbon^33^ and greening in prior studies^45,69^.

##### Drivers of seasonality

In these snow-covered areas, VIS and NIR have similar monthly variations, though with different magnitudes, which are akin to the variations of the snow cover (Fig. 4c).

#### Interactions between decreases in snow cover, increase in soil moisture, and greening

##### Drivers of long-term trends

In a number of basins in the HMA, the simultaneous influence of the changes in snow cover, soil moisture, and vegetation impacts surface albedo changes. For example, because all the three factors controlling surface albedos are preponderant, the Hwang Ho has one of the highest decreasing trends of NIR and VIS in HMA, equal to 5 10^−4^/year. In the Tarim, the decreasing trends of NIR and VIS (> − 2 10^−4^/year) are due to the decreasing trends of snow cover in winter and soil moisture and LAI from April to November (not shown here). In the Amu Darya and the other northwestern basins, the NIR has an increasing yearly trend, and the VIS has an increasing trend due to the yearly increase in LAI. The increases in VIS are also related to the decreasing trends in soil moisture. Decreases in surface albedo equal to − 4 10^−4^/year and − 8 10^−5^/year for NIR and VIS, respectively, in the central and eastern Himalayas characterized by high VIS (0.09) and low NIR (0.11) are governed by LAI, soil moisture, and snow cover. Although surface albedo is decreasing over the years, in winter it is increasing likely because greening enhances snow interception.

##### Drivers of the seasonality

**Figure 5:**
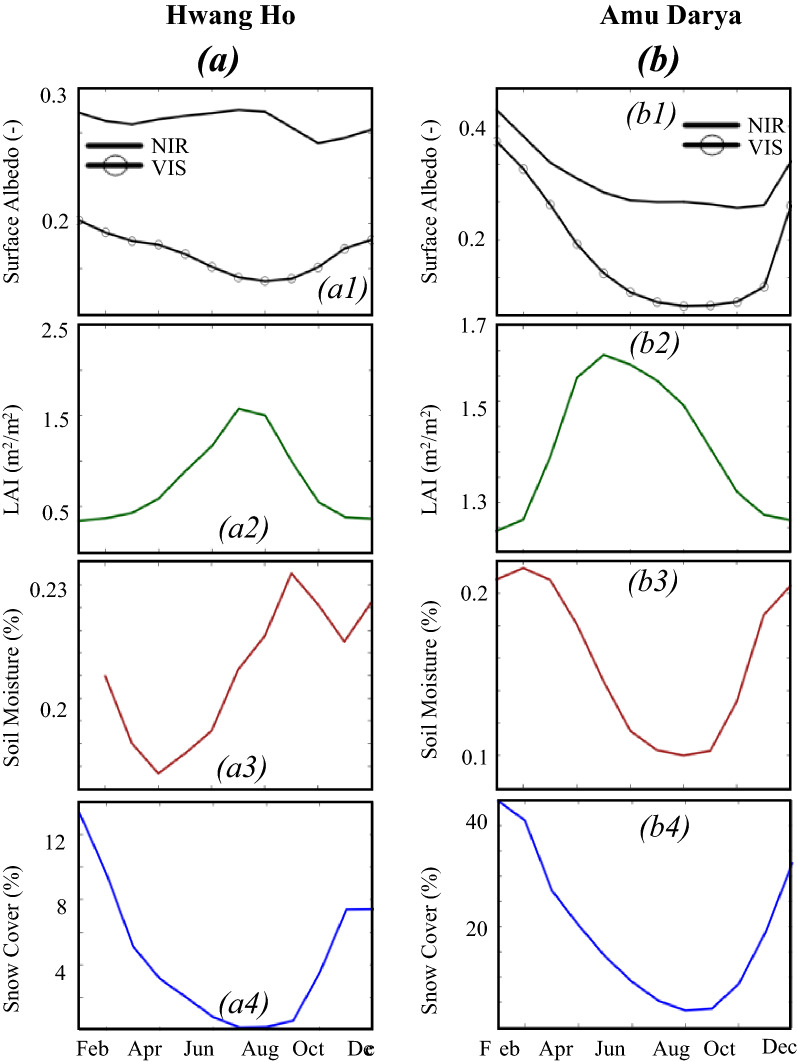
Monthly variations of the averages of surface albedo, LAI, soil moisture and snow cover in basins where surface albedo changes are controlled by greening, soil moisture, and snow cover (**a**) Hwang Ho, and (**b**) Amu Darya.

Figures 5a and b illustrate the seasonality of VIS and NIR surface albedos, LAI, soil moisture, and snow cover in the Hwang Ho and the Amu Darya. In the Hwang Ho, the VIS decreases from January to August to reach its lowest value (~ 0.08) then increases as the winter season begins contrary to the NIR (Fig. 5a). In vegetated areas where snowfall occurs, the canopy increases both VIS and NIR because the intercepted snow offsets the canopy reflectance in all wavelengths^70^. The NIR and VIS increase from January to reach their peak in March as the snow cover is high. As the canopy becomes snow-free, it starts reflecting in the NIR. As such, the decreases in NIR due to the decline of snow are compensated for by the increases induced by the canopy reflectance. Therefore, the decreases in NIR are not as sharp as in the VIS. The NIR and VIS increase again in November when the winter begins. Due to the opposite effects of snow and forests on the NIR, the second increase is only detectible in the VIS (Supplementary Fig. 2).

## Discussion

Because irrigated lands have the highest surface albedo decreases, irrigation in HMA could significantly reshape its climate dynamics. Surface albedo decreases driven by irrigation are likely to have a positive feedback impact on water resource requirements. For example, the reduction in surface albedo due to irrigation could lead to more warming and high evaporative demand, which could subsequently lead to more irrigation demand and the overuse of water resources. Over the Ganges–Brahmaputra and Indus with large populations reliant on irrigated agriculture, these surface albedo decreases are a significant concern. Another positive feedback mechanism related to cold season processes also raises concerns about the shifts in water availability. Surface albedo changes derived from the decrease in snow will further enhance this decrease in snowpack and warming. A decrease in surface albedo increases the surface absorption of solar radiation, leading to a decrease in snow and more water available for vegetation growth and, therefore, boosting greening. In snow-covered forests, on the other hand, greening increases surface albedo and could attenuate warming. The impacts of the changes in land surface features (irrigation, greening, decreases in snow) on the surface albedo will in turn accentuate these changes and the practices that have caused them. The attributions of the surface albedo changes developed in this study, therefore, are important inferences for future modeling studies for representing these interactions and feedbacks and evaluating their role in climate change. It is also important to account for this feedback in designing climate change mitigation strategies, as counterbalancing Earth’s warming could involve changes in practices such as irrigation.

## Methods

### Selected satellite-based products

We use remote sensing datasets to quantify the changes in surface albedo, LAI, soil moisture, and snow cover.

*MODIS MCD43 surface albedo* We use the surface albedos provided by NASA’s MODIS version V006 (MCD43A3) and their associated quality layers^71^. MODIS surface albedo products are generated every 8 days and have a spatial resolution of approximately 500 m. MCD43 provides BSA (directional hemispherical reflectance) which describes the albedo under direct illumination conditions in the absence of a diffuse component (i.e., when the sun as a point of source of illumination) and WSA (bihemispherical reflectance) which is defined as albedo in the absence of a direct component when the diffuse component is isotropic in NIR and VIS.

*MODIS MCD14A2H LAI* LAI, defined as the area of green leaves per unit ground horizontal surface area, is a good indicator of changes in vegetation greenness on Earth. LAI is widely used to analyze greening on Earth^72,73^. We use the LAI values provided by the MCD15A2H Version 6 of MODIS^54^ at a spatial resolution of 500 m and a temporal resolution equal to 8 days.

*MODIS Snow Cover fraction* we assess the monthly snow cover fraction estimates provided by MODIS Snow Cover fraction L3 at a spatial resolution of 0.05^55^.

*ESA CCI Soil moisture* we analyze the daily soil moisture provided by the European Space Agency Climate Change Initiative ESA CCI^56^. The ESA CCI soil moisture v05.2 consists of three surface soil moisture data sets. In this study, we use the dataset generated by blending the soil moisture retrievals from active and passive microwave remote sensing instruments.

*Irrigation* we use the dataset provided by^35^ to delineate the irrigated lands of the HMA.

*Precipitation* precipitation is highly uncertain in HMA due to data scarcity. As a result, different products derived from satellite remote sensing and reanalyses provide different results and are characterized by different spatiotemporal resolutions. To overcome these disparities, we generated a precipitation dataset using a localized probability matched method^58^ to blend three precipitation products (IMERG^59^, CHIRPS^60^, and ERA5^61^) that have been found to have the best averages and trends over HMA.

### Statistical analyses

To capture the influence of HMA heterogeneity on the surface albedo changes, we perform our analysis at 500 m, which is the spatial resolution of the surface albedo data. The changes of surface albedo and its potential control variables (LAI, snow cover, and soil moisture) over the past two decades are quantified by computing their trends using the Mann–Kendall test with a confidence level of 95%^74–76^ given by:

1 $$ S = \mathop \sum \limits_{i = 1}^{n - 1} \mathop \sum \limits_{j = k + 1}^{n} sign\left( {x_{j} - x_{i} } \right) $$

where x is the time series variable. The subscript j and k are the observation time. $sign\left( {x_{j} - x_{i} } \right)$ is equal to + 1, 0, or − 1, which means increasing, no, and decreasing trends, respectively.

Because three variables are likely controlling the changes in surface albedo, we employ the partial information decomposition framework to quantify the interactions and dependencies between these variables and the surface albedo. The partial information decomposition allows us to quantify (1) the amount of information that each control variable uniquely contributes to the surface albedo, (2) the redundant information between the three variables, and (3) the information due to the combined knowledge of the three variables, called synergistic information. More details about the computation of these metrics can be found in^75–77^. We attribute the dominant driver of the changes in surface albedo at a given point to the variable with the highest unique information. When soil moisture is the dominant driver of the changes in surface albedo and the changes in precipitation are not correlated to the changes in soil moisture and the area is irrigated, the changes in surface albedo are attributed to irrigation. Land surface processes are characterized by strong seasonality and depending on the season, the dominant factors, as well as the values of surface albedos, may change^16,19^, we, therefore, analyze the monthly variations of yearly trends and averages.

## Supplementary Information

Supplementary Information.

## Data Availability

Datasets used in this study can be found in the following websites: MODIS Albedo: https://lpdaac.usgs.gov/products/mcd43a3v006/. MODIS LAI: https://lpdaac.usgs.gov/products/mcd15a2hv006/. MODIS Snow Cover: https://nsidc.org/data/MOD10A1. ESA CCI soil moisture: https://www.esa-soilmoisture-cci.org/data. ERA5 precipitation: https://www.ecmwf.int/en/forecasts/datasets/reanalysis-datasets/era5. IMERG precipitation: https://gpm.nasa.gov/taxonomy/term/1372. CHIRPS precipitation: https://www.chc.ucsb.edu/data
