# Supplementary material for: Irrigation and warming drive the decreases in surface albedo over High Mountain Asia
Source: Sci Rep. 2022 Sep 28;12:16163. doi: 10.1038/s41598-022-20564-2 (PMC9519907; doi:10.1038/s41598-022-20564-2)
Supplement: Supplementary file 1 — Supplementary Information. [file 41598_2022_20564_MOESM1_ESM.docx]

Supplementary Information

Supplementary Figure 1: Spatial distributions of the yearly (a) averages of precipitation, LAI, soil moisture, and snow cover, (b) their corresponding annual trends from 2003 to 2020, and (c) correlation between changes in precipitation and soil moisture. Trends were computed using the Mann-Kendall test with a confidence level of 95%.

Supplementary Figure 2: Monthly variations of the averages of surface albedo, LAI, soil moisture and snow cover in the Ganges-Brahmaputra basin (a) Central and Eastern Himalayas, and (b) Snow-free forests.

Supplementary Figure 3: Monthly variations of the averages of surface albedo, LAI, soil moisture and snow cover in the (a) Irrawaddy, (b) Song Hong, and (c) Si basins.

Supplementary Figure 4: Monthly variations of the averages of surface albedo, LAI, soil moisture and snow cover in the Indus basin (a) irrigated lands, and (b) non irrigated land.

Supplementary Figure 5: Monthly variations of the averages of surface albedo, LAI, soil moisture and snow cover in the (a) Tibetan Plateau and (b) Tarim basin.

Supplementary Figure 6: Monthly variations of the averages of surface albedo, LAI, soil moisture and snow cover in the (a) Ili and (b) Syr Darya basins.

Supplementary Figure 7: Spatial distributions of the yearly trends of visible white-sky surface albedo and snow cover in March and October. Trends were computed using the Mann-Kendall test with a confidence level of 95% show increasing trends in surface albedo and snow cover in Western Himalayas in March while the surface albedo tends to decrease in October.
